# Analysis of Relations between Spatiotemporal Movement Regulation and Performance of Discrete Actions Reveals Functionality in Skilled Climbing

**DOI:** 10.3389/fpsyg.2017.01744

**Published:** 2017-10-06

**Authors:** Dominic Orth, Graham Kerr, Keith Davids, Ludovic Seifert

**Affiliations:** ^1^Centre d'Etude des Transformations des Activités Physiques et Sportives (CETAPS) - EA 3832, Faculty of Sport Sciences, University of Rouen, Rouen, France; ^2^School of Exercise and Nutrition Science, Queensland University of Technology, Brisbane, QLD, Australia; ^3^Movement Neuroscience Program, Institute of Health and Biomedical Innovation, Queensland University of Technology, Brisbane, QLD, Australia; ^4^Centre for Sports Engineering Research, Sheffield Hallam University, Sheffield, United Kingdom

**Keywords:** affordances, exploration, functional movement variability, rock climbing, motor skill, skill transfer

## Abstract

In this review of research on climbing expertise, we focus on different measures of climbing performance, including spatiotemporal measures related to fluency and activity states (i.e., discrete actions), adopted by climbers for achieving overall performance goals of getting to the end of a route efficiently and safely. Currently, a broad range of variables have been reported, however, many of these fail to capture how climbers adapt to a route whilst climbing. We argue that spatiotemporal measures should be considered concurrently with evaluation of activity states (such as reaching or exploring) in order gain a more comprehensive picture of how climbers successfully adapt to a route. Spatial and temporal movement measures taken at the hip are a traditional means of assessing efficiency of climbing behaviors. More recently, performatory and exploratory actions of the limbs have been used in combination with spatiotemporal indicators, highlighting the influence of limb states on climbing efficiency and skill transfer. However, only a few studies have attempted to combine spatiotemporal and activity state measures taken during route climbing. This review brings together existing approaches for observing climbing skill at performance outcome (i.e., spatiotemporal assessments) and process (i.e., limb activity states) levels of analysis. Skill level is associated with a spatially efficient route progression and lower levels of immobility. However, more difficult hold architecture designs require significantly greater mobility and more complex movement patterning to maintain performance. Different forms of functional, or goal-supportive, movement variability, including active recovery and hold exploration, have been implicated as important adaptations to physiological and environmental dynamics that emerge during the act of climbing. Indeed, recently it has also been shown that, when climbing on new routes, efficient exploration can improve the transfer of skill. This review provides new insights into how climbing performance and related actions can be quantified to better capture the functional role of movement variability.

## Introduction

Traditionally, skilled climbing is characterized by the efficiency of spatial and temporal patterns that emerge at the center of mass (COM) during the act of climbing (Billat et al., 1995; Cordier et al., 1996). Temporal assessment quantifies the number and nature of stoppages relative to continuous climbing, indicating the amount of time spent in isometric contraction (Billat et al., 1995; White and Olsen, 2010). Spatial indicators highlight the efficiency of a climber's trajectory across the surface, estimating the ability to perceive an efficient “pathway” through the route (Cordier et al., 1993; Boschker and Bakker, 2002). Finally, combined spatiotemporal measures, such as the minimization of jerk, globally indicate how smoothly climbing movements are linked together (Seifert et al., 2014b). Importantly, evaluating performance along spatial and temporal variables can address different mechanisms underpinning skilled climbing (Cordier et al., 1996). For example, initial and rapid improvement in performance is believed to be primarily influenced by the rapidly adapting visual-motor system (Pezzulo et al., 2010). Alternatively, a climber may improve performance by linking movements in a more periodic fashion. These sorts of improvement occur over longer time-scales, such as the months and years required for musculoskeletal system adaptation (Vigouroux and Quaine, 2006).

More recently, activity states such as reaching and grasping have been distinguished as having exploratory (information gathering) or performatory (body progressing) qualities, providing an estimate of the intentions underpinning an individual's actions during climbing (Pijpers et al., 2006). For example, changing task constraints, such as height from the ground during climbing practice (Pijpers et al., 2006), does not physically modify climbing affordances. Where climbing affordances are defined as opportunities for qualitatively distinct actions that support climbing such as hold reachability, grasp-ability, stand on-ability and specific climbing movements (Boschker et al., 2002). However, increasing climbing height can interact with an individual's emotional state. This can alter the discrete actions used during climbing, transiently, on the basis of altered intentions brought about by an increased state of anxiety. In this case, changes in emotional states can influence *intentions* toward information pick-up for remaining fixed to the wall, as opposed to achieving vertical progression. Inferences of climbers' intentions are generally based on behavioral data. For example, when an individual reduces the distance they are willing to reach for grasping holds, or they increase their use of exploratory actions, this suggests the climber is primarily concerned with stability as opposed to efficient progression (Pijpers et al., 2006; Seifert et al., 2014c).

A limitation in the extant literature is poor understanding of how an individual's specific activity state can influence climbing efficiency and being able to combine these measures can be highly informative (Orth et al., 2016). Indeed, approaches that have considered these variables in combination have uncovered important insights into the functional or goal-supportive characteristics of movement variability (Fryer et al., 2012; Seifert et al., 2015). For instance, Pijpers et al. (2006) implied that exploratory behavior reflects poor performance. More recent studies have combined the analysis of exploratory actions with spatiotemporal performance outcomes, revealing that exploratory actions can be related to an improvement in performance through practice (Seifert et al., 2015). Thus, this review draws together studies that have reported on performance and discrete limb actions in climbing tasks to evaluate how, in combination, these analyses can explain successful and efficient climbing. The review is structured into three parts. First, we examine the existing state of the art on how spatial and temporal outcomes are used to quantify skilled climbing. Next, data pertaining to activity states are considered with respect to their functionality for the individual. Finally, hypotheses are presented for how activity states combined with spatial-temporal outcomes can indicate specific intentions of climbers during the act of climbing.

## Search methodology

Medline and SPORTDiscus databases were searched for published primary sources. Keywords related to climbing (rock climbing, ice climbing, mountain climbing, boulder climbing, artificial climbing, top-rope climbing, lead-rope climbing, mixed climbing, indoor climbing, outdoor climbing, route climbing, slope climbing) were pooled (via Boolean operation “OR”) and combined (via Boolean operation “AND”) with keywords related to skilled behavior (skill, transfer, perform, ability, expert, novice, beginner, intermediate, advanced, elite, dynamic, force, kinematics, kinetics, perception, action, cognition, behavior, center of mass, trajectory, movement, movement pattern, recall, gaze, vision, coordination, motor, feet, hand, foot, grasp, reach, pattern, intervention, pedagogy, feedback, constraint, coach, learn, practice, applied, train, fluency, fluidity, smoothness, jerk, activity state, classification, intention, exploration, strategy) and also pooled via Boolean operation “OR.” Results were limited to human participants, written in the English language, and, Medline and SPORTDiscuss databases searched from their earliest available record up to November 2016. Google Scholar was then used to scrutinize the related articles and referencing studies. Reference lists of all eligible studies were then manually inspected.

Articles were restricted to those written in the English language. Restrictions were also made on the participant sample, study design and outcomes measures. Specifically, for inclusion, studies were required to report sample characteristics so that ability level could be estimated as either beginner, intermediate, advanced, elite or upper elite (Draper et al., 2011a). Study designs were limited to experimental or technical reports that involved climbing a surface graded for difficulty (Draper et al., 2011a). Furthermore, studies where the task goal did not, implicitly or otherwise, require getting to the end of the route were excluded. For example, if the task required participants to adopt a static posture or perform isolated reach and grasp actions, it was excluded since such task constraints do not impose a route finding problem. Outcomes were restricted to at least one measure to quantify spatial, temporal patterns of the COM or limbs, or, activity state during actual climbing. Appraisal of article quality was evaluated in terms of potential contribution to understanding how activity states influence performance efficiency along spatial-temporal measures. Eligible experiments were then identified to a standardized form which was then used to extract relevant study data (see Table 1). These included: experimental design, sample characteristics, interventions (including detailed characteristics of route design properties), task characteristics, independent variables and levels, outcome measures, and comparisons and interaction effects.

**Table 1:** Studies fulfilling inclusion criteria.

**Study^a^**	**Sample^b^**	**Design^c^**	**Task^d^**	**Measure^e^**	**Outcome^f^**
**SPATIAL**					
Boschker and Bakker (2002) [MMD] [Journal article]	*N* = 24,18–28 yrs, no experience: control subgroup (n = 8); dual grasping model subgroup (n = 7); arm-crossing technique model subgroup (*n* = 9)	A. Pedagogical intervention (model) i. control (observed the climbing wall) ii. simple technique model (observed an expert climber 4 times using a basic climbing technique) iii. advanced technique model (observed an expert climber 4 times using an advanced climbing technique) B. Practice (t × 5) [note: all observations were on a video, when observing the expert model, playback speed was first in slow motion (x2) and then normal (x2)]	Climb (indoor, artificial, top-roped, F-RSD = 5c [1, Intermediate], crux = 1,7 m height, 3.5 m width, 98.2 deg relative to floor, 22 holds) instructed to climb using the same technique as observed model otherwise self-preferred	Movement (hip trajectory, discrete actions) single camera: 1. GIE 2. falls [climb time]	At trial 2, 3, and 4, the advanced technique subgroup climbed significantly faster than the control and simple technique subgroup; At trial 1, 1 was significantly lower in the advanced technique subgroup compared to the simple technique subgroup and significantly lower in the control subgroup compared to the simple technique subgroup; At trials 2, 3 and 4, 1 was significantly lower in the advanced technique subgroup compared to the control and simple technique subgroups
Cordier et al. (1993) [MMD] [Journal article]	*N* = 7: average skill subgroup (*n* = 3, F-RSD = 6b-6c [1.75–2.25, Intermediate]); highly skilled subgroup (*n* = 4, F-RSD = 7a-7b [2.5–3, Intermediate-, Advanced])	A. Skill B. Practice (*t* × 10)	Climb (indoor, artificial, top-roped, F-RSD = 6a [1.25, Intermediate], ~10 m high) self-preferred	Movement (hip trajectory) single camera: 1. GIE 2. fractal dimensions [climb time]	1 was significantly lower in highly skilled subgroup; 1 significantly decreased with practice in both groups; [note: a significant interaction effect between skill and practice showed that 1 reduced faster in the higher skilled subgroup compared to the lesser skilled subgroup; a clear correlation was shown between climb time and entropy with higher climb times being associated with higher entropy]
Cordier et al. (1994b) [MMD] [Journal article]	Average skill subgroup (F-RSD = 6b [1.75, Intermediate]); highly skilled subgroup (F-RSD = 7b [3, Advanced]) [note: the exact number of individuals making up each sub-group not reported]	A. Skill B. Practice (*t* x 10)	See above, Cordier et al. (1993)	Movement (hip trajectory) single camera: 1. GIE [climb time]	Highly skilled subgroup showed less 1 compared to the average skilled subgroup; with practice 1 significantly reduced; highly skilled subgroup reduced entropy faster with practice than the skilled group; [note: highly skilled subgroup reduced entropy to asymptote by trial three whereas the average skill subgroup did not reach a clear asymptote after 10 trials of practice]
Cordier et al. (1994b) [MMD] [Journal article]	*N* = 10: non-expert subgroup (*n* = 5, F-RSD = 6b [1.75, Intermediate]); expert subgroup (*n* = 5, F-RSD = 7b [3, Advanced])	A. Skill B. Practice (*t* × 10)	See above, Cordier et al. (1993)	Movement (hip trajectory) single camera: 1. GIE [climb time]	Highly skilled subgroups showed overall less entropy compared to the average skilled subgroup; With practice entropy significantly reduced; Highly skilled group reduced entropy faster with practice than the average skilled group; Highly skilled group reduced entropy to asymptote by trial three. Unskilled group did not appear to reach asymptote.
Pijpers et al. (2003) [RM] [Journal article] – Experiment 2	*N* = 17, 11 M, 19–26 yrs, little to no experience in climbing	A. Route design (height) i. mean height of foot holds 0.3 m from the ground ii. foot holds 3.7 m from the ground	Climb (indoor, artificial, top-rope, flush vertical, 6 hand- and 5 foot-holds, 7 m height, 3.5 m width) nr [note: difficulty assumed as easily achievable; participants practiced on route before testing; each trial required 20 sec continuous climbing]	Movement (hip trajectory) single camera: 1. GIE [climb time, HR and state anxiety]	1 and climb time significantly increased when climbing in the high condition
Sanchez et al. (2010) [IG] [Journal article]	*N* = 19, 24.6 yrs ± 4.0 SD, elite climbers, F-RSD = 7b+ to 8b [3.25–4.5, Advanced-Elite]: successful subgroup (n = 9); unsuccessful subgroup (n = 7) [note: successful subgroup membership criteria required that the climbers get to at least the 39th hold (out of 50). Those who did not were assigned to the unsuccessful subgroup.]	A. Skill	Climb (artificial, F-RSD = 7c+ [3.75, Advanced]], crux = 2, rest points = 2, on-sight, 16 m high, 50 handholds) competition [preview = 5 mins]	Movement (hip trajectory) single camera: 1. GIE (section 1 crux, section 1, section 2) 2. climb time (section 1 crux, section 1, section 2) [precompetitive state anxiety] [note: 16/19 of the climbers were analyzed; for analysis the route was broken into 2 sections and 2 crux points]	2 was significantly longer in the successful subgroup compared to the unsuccessful subgroup in the first crux.
Zampagni et al. (2011) [IG] [Journal article]	*N* = 18 M: elite subgroup (*n* = 9, 32.1 yrs ± 7.6 SD, F-RSD = 7b-8b [3–4.5, Advanced-Elite], climbing age = 13.9 yrs); no experience subgroup (*n* = 9, 31.9 yrs ± 8.5 SD)	A. Skill	Climbing (artificial, top-rope, 20 holds, uniform holds = 13 cm high, 16 cm wide, 12 cm deep) under instruction [note: instructed on the sequence of which limb to reposition and to which hold, this pattern was repeated until climbers reached the top; climbers were required to complete each cycle within 4 s]	Movement, applied force (COM, hands and feet) mulit-camera, instrumented holds: 1. COM anterior/posterior and lateral motion (min, mean, max) 2. force (vertical component)	The expert subgroup climbed with 1 significantly further from the wall and with larger lateral displacements compared to the no experience subgroup; 2 showed significantly larger oscillations in the expert subgroup compared to the no experience subgroup.
**TEMPORAL**					
Billat et al. (1995) [RM] [journal article]	*N* = 4, 22.2 yrs ± 2.3 SD, F-RSD = 7b [3, Advanced], climbing age = 3 yrs	A. Hold (size) and Wall (slope) i. smaller more complex hold design ii. steeper slope [note: difficulty matched]	Climb (indoor, artificial, F-RSD = 7b [3, Advanced], red-point, 15 m high, ~10 deg overhang) self-preferred [note: 5 hrs practice on each route prior to testing]	Movement (discrete actions) single camera: 1. dynamic time (discernable motion at the hips) 2. static time (no discernable motion at the hips) [note: additional variables of interest related to oxygen consumption]	1 was significantly longer on the smaller more complex route compared to the route with a larger overhang.
Cordier et al. (1996) [MMD] [Journal article]	*N* = 10: non-expert subgroup (*n* = 5, F-RSD = <7a [ <2.5, Intermediate]); expert subgroup (*n* = 5, F-RSD > 7a [>2.5, Advanced])	A. Skill B. Practice (*t* × 10)	See above, Cordier et al. (1993)	Movement (hip trajectory) single camera: 1. frequency of movement (Hz) 2. harmonic analysis	Expert subgroup generated approximately one movement every three seconds and were closer to the harmonic model by a factor of about two compared to the non-expert subgroup
Draper et al. (2011b) [MMD] [Journal article]	*N* = 18, 12 M, 25.6 ± 4.5 intermediate level, onsight lead F-RSD = 5+ [1, Intermediate], red-point F-RSD = 6a [1.25, Intermediate] climbing age 3.6yrs ± 3.1	A. Route Type i. tope-rope ii. lead rope B. Route completion i. yes (*n* = 11) ii. no (*n* = 7) [note: group formed post hoc based on those who did or did not fall]	Climb (indoor, artificial, F-RSD = 6a, 12.5 m height, 7 quick-draws) self-preferred	Movement (climb time) single-camera [yrs experience, NASA-TLX, CSAI-2D, oxygen consumption, blood lactate, HR] 1. climb time (between successive quick-draws)	Experience was the best predictor of climbing success and was also correlated with confidence and faster climbing within challenging parts of an ascent. Climbers that fell were slower through the route
White and Olsen (2010) [Journal article] [RM]	*N* = 6, elite, age = 28yrs ±5 SD, climbing age = 16yrs ± 5SD [note: sample argued elite, held an IFSC World ranking for the World Cup boulder series and members of British national team]	Observational	Climb (indoor, artificial, bouldering) competition [a total of 12 climbs were recorded, two climbs per individual, each on a different route]	Movement (discrete actions) two-cameras: 1. hand contact time 2. reach time 3. dynamic time 4. static time [number of attempts, climb time, total attempt time, between attempt recovery time]	A larger proportion of time is spent in dynamic movement relative to static. Hand contact time was larger than reach time
**ACTIVITY ANALYSIS**					
Nieuwenhuys et al. (2008) [RM] [Journal article]	*N* = 12, 7 M, 24.4 yrs ± 1.98 SD, no experience	A. Route design (height) i. holds 0.44 m from the ground ii. holds 4.25 m from the ground	Climb (indoor, artificial, top-rope, 26 hand- and foot-holds) self-preferred [note: difficulty level assumed to be easily achievable; participants practiced on the route prior to testing]	Visual behavior, movement (gaze-location, discrete actions) eye-tracker, single camera; 1. fixation (duration, number, average duration, duration per location, duration per type, search rate) [note: possible fixation locations included handholds, hands, wall, other and possible fixation types were exploratory or performatory] 2. mean distance of fixation 3. movement time (climb time, stationary time, moving time (hands and feet), average movement duration between holds) 4. mean distance of hand movements [nb: additional measures of interest were HR and anxiety]	Climb time, movement time between holds and time spent static was significantly longer and number of movements were significantly greater in the high condition compared to the low condition; Fixation durations were significantly longer, number of fixations significantly increased, and search rate significantly decreased in the high condition compared to the low condition.
Pijpers et al. (2005) [RM] [Journal article] – Experiment 1	*N* = 8 M, 31.4 yrs ± 4.81 SD, no experience	A. Route design (height) i. mean height of foot holds 0.4 m from the ground ii. foot holds 5.0 m from the ground	Climb (indoor, artificial, top-rope, flush vertical, flash, 7 m height, 3.5 m width, 7 hand- and 6 foot-holds, mean inter-hold distance = 0.15 m) as fast and as safely as possible without falling: [note: difficulty not given but assumed to be easily achievable; participants practiced on low traverse prior to testing and observed an expert model perform the traverse on video; each trial required 2 traversals]	Movement (discrete actions) multi-camera: 1. number of exploratory movements (number of times a hold is touched without use as support) 2. number of performatory movements 3. Use of additional holds (two holds not needed to achieve traversal were set into the route) [climb time, HR and anxiety data]	1 and climb time was significantly higher in the high condition compared to the low condition
Pijpers et al. (2006) [RM] [Journal article] – Experiment 2	N = 12, 6 F, 20.8 yrs ± 3.57SD, no experience	A. Route design (height) i. holds on average 0.36 m from the ground (*t* x 4) ii. holds 3.69 m from the ground (*t* x 4)	Climb (indoor, artificial, top-rope, flush vertical, 7 m height, 3.5 m width, 15 hand- and 15 foot-holds) as fast and as safely as possible without falling [note: difficulty not rated but assumed to be easily achievable; participants practiced on route before testing; each trial required 2 traversals]	Movement (discrete actions) single camera: 1. number of performatory actions (hands and feet) 2. number of exploratory actions (hands and feet) [climb time, state anxiety]	1, 2 and climb time increased significantly when climbing at height compared to close to the ground
**CROSSED**					
Fryer et al. (2012) [IG] [Journal article]	*N* = 22: intermediate subgroup (*n* = 11, 7 M, F-RSD = 6a/ Ewbank = 18/19 [1.25, Intermediate], climbing age = 3 ± 1.15 yrs); advanced subgroup (*n* = 11, 10 M, F-RSD = 6c+/ Ewbank = 21/22 [2.25, Advanced], climbing age = 3.3 ± 1.06 yrs)	A. Skill	Climb (indoor, artificial, top-roped, F-RSD = 6a [1.25, Intermediate] and 6c+ [2.25, Intermediate], on-sight, 12.15 m high, overhang) self-preferred [preview = 5 min] [note: difficulty matched to subgroup skill levels]	Movement (discrete actions) single camera: 1. time spent static (no hip motion) 2. time spent actively resting (shaking the limbs) [note: additional variables of interest related to HR, mood state, anxiety]	Advanced subgroup spent significantly greater proportion of their climb time in static states and more of the static time actively resting compared to the intermediate subgroup; [note: significantly lower heart rates in the advanced subgroup compared to the intermediate subgroup are interpreted as related to the time spent in active recovery]
Pijpers et al. (2005) [RM] [Journal article] – Experiment 2	*N* = 15, 13 M, 20.7 ± 2.22 SD yrs, no experience	A. Route design (height) i. mean height of foot holds 0.4 m from the ground ii. foot holds 4.9 m from the ground	Climb (indoor, artificial, top-rope, flush vertical, 7 m height, 3.5 m width, 6 hand- and 5 foot-holds) as fast and as safely as possible without falling: [note: difficulty not given but assumed to be easily achievable; participants practiced on low traverse prior to testing and observed an expert model perform the traverse on video; each trial; 4 traversals required per condition]	Movement (discrete actions) mulit-camera, instrumented holds: 1. number of exploratory movements 2. number of performatory movements (hands and feet) 3. rest between traversals 4. contact time (total, hands, feet, average per hold, total and for feet and hands) [climb time, HR, anxiety]	1 and 2 (feet only) was significantly greater and 4 (total, feet and hands, average total and average feet) was significantly longer in the high condition compared to the low condition. [note: climb time was significantly longer in the high condition compared to the low condition]
Sanchez et al. (2012) [MMD] [Journal article]	*N* = 29: intermediate subgroup, (*n* = 9, F-RSD = 6a – 6b [1.25–1.75, Intermediate]); advanced subgroup, (n = 9, F-RSD = 7a-7a+ [2.5–2.75, Intermediate-Advanced]), expert subgroup, (*n* = 11, F-RSD > 7b+ [>3.25, Advanced])	A. Skill B. Preview: i. with preview (3 min) ii. without preview	Climb (indoor, top-rope, on-sight) self-preferred [preview = 3 minutes (when given)] [note: a total of 6 routes were involved, route difficulties as follows: i. 2 intermediate routes (6a [1.25, Intermediate], 6a+[1.5, Intermediate]) ii. 2 advanced routes (both 6c [2.25, Intermediate]) iii. 2 expert routes (7b, 7c [3.5, Advanced]); participants only climbed routes that were either equal to or less than their F-RSD level]	Movement (discrete actions) single camera: 1. number of movements (performatory and exploratory) 2. duration of movements (performatory and exploratory) 3. number of stops (appropriate and inappropriate) 4. duration of stops (appropriate and inappropriate)	3 (appropriate) and 4 (appropriate) were significantly longer when climbing without preview in the expert subgroups compared to the intermediate and advanced subgroups on the route matched to skill level.
Seifert et al. (2013b) [IG] [Journal article]	*N* = 15 M: expert subgroup (*n* = 7, 32.1 yrs ± 4.0 SD, F-RSD = 7a+ to 7c [2.75–3.5, Advanced], F-RSD for ice falls = 6–7, rock-climbing age = 17.1, ice climbing age = 10.4 yrs); beginner subgroup (*n* = 8, 28.5 yrs ± 6.4 SD, climbing age ~ 20 hrs practice on artificial walls, no experience in ice climbing)	A. Skill	Climb (outdoors, ice fall, 85 deg ramp, 30 m high, top-rope) self-preferred [note: Route difficulty: i. grade 5+ (F-RSD for ice-falls) ii. grade 4 (F-RSD for ice-falls); participants only climbed routes that were equal to their F-RSD level]	Movement (upper and lower body) single camera: 1. exploration index (ratio of ice tool swings to definitive anchorages for upper and lower limbs) 2. relative angular position (upper and lower limbs pairs relative to the horizontal)	1 showed a 1:1 ratio in the expert subgroup for both the upper and lower limbs whereas 1 showed a ratio of 0.6 and 0.2 in the upper and lower limbs respectively in the beginner subgroup (i.e., more non performatory movements); 2 showed more variability in the relative angular positions in the expert subgroup compared to the novice subgroup.
Seifert et al. (2014a) [Journal article]	*N* = 8, 21.4 yrs ± 2.4SD, top-rope, F-RSD = 6a [2, Intermediate], climbing age = 4.1yrs ± 2.1 SD	A. Route design (holds) i. single edged (all edges parallel to ground) ii. double edged (one edge parallel to ground, one edge perpendicular to ground) B. Practice (4 trials)	Climb (indoors, artificial, top-roped, on-sight and practice, F-RSD = 5c [1, Intermediate] 10 m height, 20 holds, preview = 3 mins) self-preferred [note: each hold had two graspable edges]	Movement (hip) worn sensor 1. jerk coefficient (normalized) [note: rotation and position analysis] 2. Exploratory movements	1 was higher on double edged (more complex) route. 1 decreased with practice. 2 decreased with practice. [note: of additional interest was the strong correlation between rotational and positional coefficients of jerk]
Seifert et al. (2013a) [IG] [Journal article]	*N* = 15, 24.5 yrs ± 4.5 SD, naïve ice climbers: novice subgroup (*n* = 10, F-RSD <5 [<0.75, Lower grade], climbing age = 10 hrs practice on artificial walls); intermediate subgroup (n = 5, F-RSD = 6a [<1.25, Intermediate], climbing age = 3 yrs)	A. Skill [note: research question of interest was whether skill influenced transfer to different environmental properties based on the climbers history. IV corresponds to: B. Transfer i. rock climbing; ii. ice climbing.]	Climb (outdoors, ice, 30 m high, top-rope, route F-RSD for ice falls = 4) self-preferred	Movement (discrete actions) single camera: 1. exploration index (ratio of ice tool swings to definitive anchorages for upper and lower limbs) 2. relative angular position (upper and lower limbs pairs relative to the horizontal) 3. relative phase (upper and lower limb pairs) [note: see note in Seifert et al. (2011)] 4. vertical distance climbed in 5 mins 5. plateau duration (plateau defined as less than 0.15 m of vertical displacement for longer than 5 s)	1 was closer to a ratio of one swing to one definitive anchorage for intermediate subgroup compared to the novice subgroup; 2 and 3 showed significantly greater variability in the intermediate subgroup compared to the novice subgroup; 4 was significantly greater and 5 was significantly shorter in the intermediate subgroup compared to the novice subgroup. [note: of additional interest in this study was to undertake an unsupervised hierarchical cluster analysis using the DVs to classify the climbers into different skill based subgroups
Seifert et al. (2014b) [IG] [Journal article]	*N* = 14; expert climber subgroup (*n* = 7, 32.1 ± 6.1 SD, F-RSD for rock = 7a ± 7c [2.75–3.5, Intermediate], F-RSD for icefalls = 6–7, climbing age = 17.4 yrs ± 5.6); beginner subgroup (n = 7, 29.4 yrs ± 6.8, climbing age = <20 hrs indoor climbing practice)	A. Skill	Climb (outdoors, ice, top-rope; 30 m high) self-preferred [note: a total of 2 routes were involved, the expert subgroup were tested on a grade 5+ (F-RSD for ice-falls); the beginner subgroup were tested on a grade 4 (F-RSD for ice-falls)]	Movement, verbalization (discrete actions, self-confrontation interview) single camera, audio: 1. number and duration of stops 2. relative angular position (upper and lower limbs pairs relative to the horizontal) 3. exploratory and performatory actions 4. verbalisations i. perceptions ii. actions iii. intentions	Expert subgroup achieved greater vertical displacement, had more stoppages but that were shorter in duration, explored a larger angular range with ice-tools, less exploratory actions compared to beginner subgroup. Expert subgroup verbalized about information related to behavioral opportunities that were multi-modal and intentions were focused on vertical traversal. Beginners focused on visual cues for putting their ice-hooks into the wall and focused intentions on remaining on the wall
Sibella et al. (2007) [RM] [Journal article]	*N* = 12, 30.6 yrs 16–49, recreational, non-competitive climbers, training 1–2 x per week: agility style climber subgroup (n = 1); force style climber subgroup (n = 1)	A. Skill [note: skill groups formed *post hoc*, by identifying different climbing strategies using kinematic measures]	Climb (indoor, artificial, top-rope, F-RSD = 4b [0.25, Lower grade], 3 m traverse, 3 m ascent) self-preferred [note: t x 5, data averaged across participants]	Movement (COM) multi-camera: 1. GIE [note: computed for frontal, sagittal and transverse planes] 2. absolute velocity (COM) 3. absolute acceleration (COM) 4. power of acceleration time course (COM) 5. mean number of holds in contact per recorded frame of video (60 hz)	1 was significantly lower (frontal and sagittal planes), 3 and 4 was significantly lower, and 5 was significantly higher in the agility style climber compared to the force style climber; 2 was significantly lower in the agility style climber compared to the entire group of climbers and 2 was significantly higher in the force style climber compared to the entire group of climbers

a *Author (date) [experimental design] publication type*.

b *Sample size; (sample characteristics: age, variability, climbing age, reported ability level [ability level converted to Watts]); subgroups*.

c *Independent variable: A, B; level: i, …, iii*.

d *Task, climb; (route properties: location (indoors; outdoors), wall properties (artificial; rock; ice, height, slope), type (top-rope; lead), route difficulty [Watts conversion (see^b^)]; instructions; [preview time]*.

e *Dependent variable type; (level or nature of analysis); measurement device; dependent variable 1, …, 5 (description and sub-levels) [additional variables]*.

f *Variable(s) reported showing significant effect: 1, …, 5 (description of direction of effect and reported interpretation as position or negative for performance)*.

*COF, coefficient of friction; COP, center of pressure; CPEI, climbing performance evaluation inventory; CRP, continuous relative phase; Crux, a part of a route more difficult than others; deg, degrees; DV, dependent variable; F, female; flash, individuals have had a chance to observe another climber on the route prior to making an attempt; F-RSD, french rating scale of difficulty; IG, Independent groups; IV, independent variable; GIE, geometric index of entropy; HR, heart rate; hrs, hours; Hz, cycles per second; M, male; m, meters; max, maximum; min, minimum; mins, minutes; MMD, mixed methods design; NASA-TLX, National Aeronautics and Space Administration Task Load Index; nr, not reported; on-sight, the first attempt of a climbing route; PCA, Principle component analysis; red point, refers to performance on a route that has been previously practiced; s, seconds; SD, standard deviation; t, trials; UIAA, Union Internationale des Associations d'Alpinisme; vs., versus; yrs, years*.

## Results

Using the search methodology, the Medline database yielded 1,099 titles and abstracts. These were screened yielding 35 relevant articles, which were identified and their full texts retrieved. Relevant studies were then screened using the standardized inclusion criteria and 13 eligible studies identified. Using the same search methodology, the SPORTDiscsuss database was searched. This analysis yielded 2,201 results from which titles and abstracts were screened, and 59 relevant articles identified. After duplicate removal, full texts were retrieved and eligibility was assessed using the standardized inclusion criteria, identifying 15 studies this way. The related articles, citing articles and reference lists of 7,400 eligible studies were searched using Google Scholar. 94 relevant studies were subsequently identified for eligibility screening. After duplicates were removed, an additional 13 eligible studies were identified this way. The article search was stopped at this point. From this pool of 41 studies, 21 fulfilled the eligibility criteria. These are summarized to Table 1 and form the basis of the discussion below.

## Spatial and temporal measures of skilled adaptation to route properties in climbing

Data on skilled climbing behavior can reflect coordination of actions to route properties, providing insights on the quality of movement adaptations. A number of studies have incorporated spatial and temporal measures into a single outcome to quantify climbing fluency. These have generally involved the analyses of the climbers' COM projection, to estimate velocity (Cordier et al., 1996; Sibella et al., 2007), acceleration (Cordier et al., 1996; Sibella et al., 2007), jerk (Seifert et al., 2014b), and phase portrait patterning (Cordier et al., 1996). Among these, linked to the number sub-movements used in carrying out an action (Elliott et al., 2010), jerk coefficients on hip movements provide the most straightforward indication of capacity to co-adapt spatial-temporal demands of performance (Seifert et al., 2014b). For example, Seifert et al. (2014b) calculated jerk coefficients on three dimensional hip translation and rotation accelerations. Here, jerk coefficients improved with practice on a route that involved use of different types of grasping techniques (overhand grasping and pinch grips), compared to no significant change on a route that required use of a single type of action (overhand grasping) (Seifert et al., 2014b).

Whilst expertise in climbing involves highly adaptive and proficient performance along both spatial and temporal dimensions in combination, current understanding of skill and practice effects has been primarily approached by considering each dimension separately (Cordier et al., 1996; Sibella et al., 2007).

### Spatial indicators of climbing fluency

Spatial indicators relate to analyses of displacement on a surface. Existing approaches include computation of the geometric index of entropy (GIE, see equation 1 below) (Cordier et al., 1993, 1994a,b; Boschker and Bakker, 2002; Pijpers et al., 2003; Sibella et al., 2007; Sanchez et al., 2010; Seifert et al., 2015; Watts et al., 2016; Orth et al., 2017), climb distance (Green and Helton, 2011; Seifert et al., 2013b, 2014c; Green et al., 2014), average movement distance (Nieuwenhuys et al., 2008), COM-to-wall distance (Zampagni et al., 2011), and planar displacement of the COM (Zampagni et al., 2011). Interpreting the quality of displacement with respect to a route is the main reason GIE has enjoyed widespread application (Cordier et al., 1993, 1994a,b; Boschker and Bakker, 2002; Pijpers et al., 2003; Sibella et al., 2007; Sanchez et al., 2010; Seifert et al., 2017).

Specifically, GIE is given for a given trajectory *x* : [*O, T*] → *R*^3^, letting Δ*x* be the trajectory length (Equation 1) and Δ*c*(*x*) the convex hull parameter. The GIE is given by:

(1) $$\begin{array}{l} \Delta x\;=\sum_{i=1}^{N}\sqrt{x_{i}^{2}+y_{i}^{2}} \end{array}$$

(2) $$\begin{array}{l} GIE_{x}=\frac{\log\left(2\;*\;\Delta x\right)-\log\left(\Delta c\left(x\right)\right)}{\log\left(2\right)} \end{array}$$

**Figure 1:**
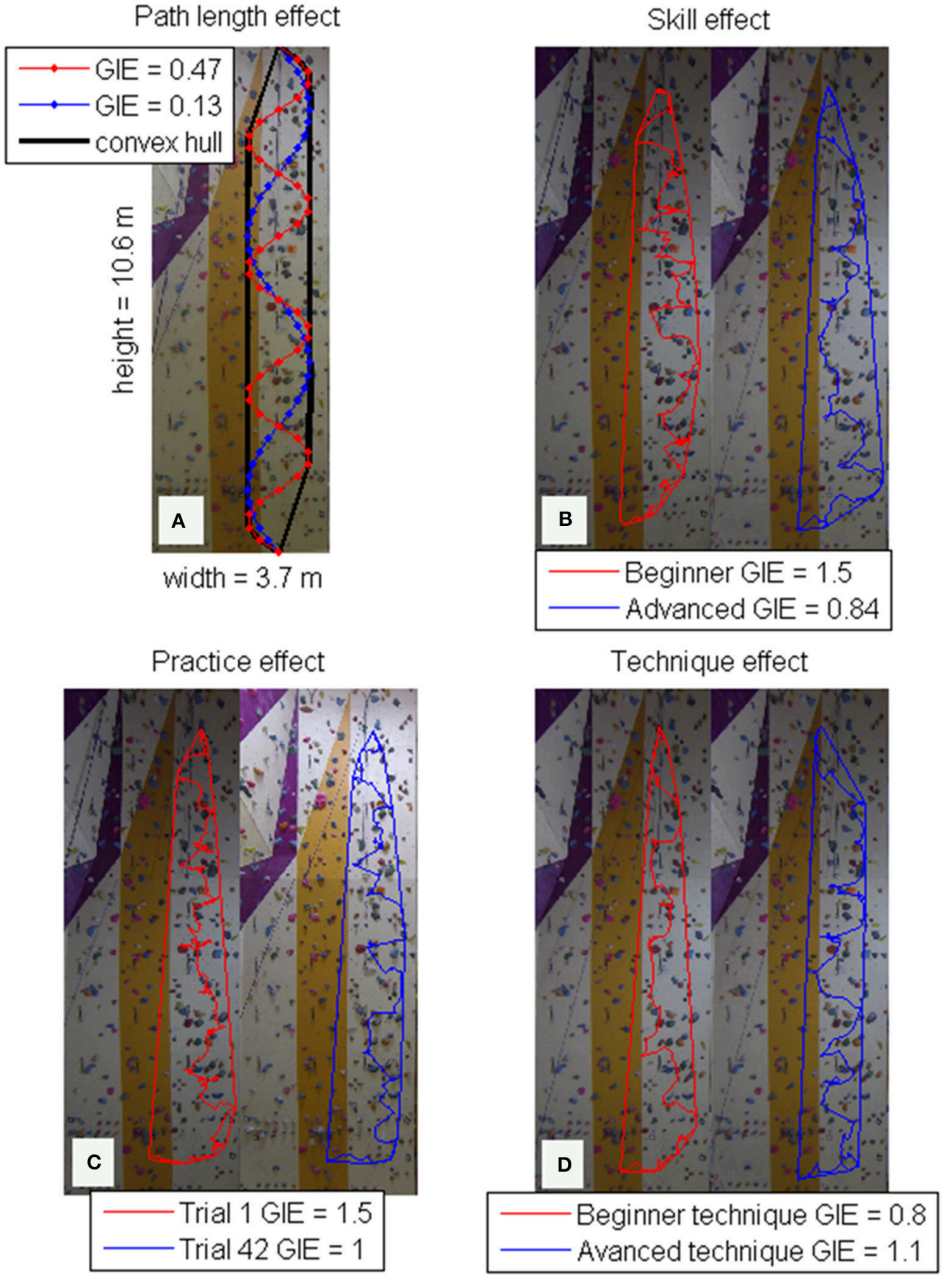
**(A)** Shows that the shorter the path length within a given convex hull, the lower the geometric index of entropy (GIE). **(B)** The blue line is data from an advanced climber, who shows a more straight forward trajectory (and thus lower GIE) compared to the beginners climbed trajectory (red line). **(C)** After practice (the blue line representing a climbed trajectory after 42 trials of practice) typically do not show periods of searching as shown in the first trial of practice (red line on the left). **(D)** When the technique used is more complex, GIE also increases. Here an individual was asked to climb the same route either with the front of the body remained facing the wall (red line) or with the side of the body facing the wall (blue line). The more advanced technique required an increase in movement complexity.

According to Cordier et al. (1994b) the GIE can assess the amount of fluency of a curve. The higher the entropy value, the higher the irregularity of the climbing trajectory, whereas the lower the entropy value, the more regular is the climbed trajectory. GIE has a number of advantages over reported spatial variables, such as the average movement distance (Nieuwenhuys et al., 2008), in that it is based on theoretically generalizable principles (Cordier et al., 1994b), readily interpreted with respect to climbing activity, and, is effective for detecting skill (Cordier et al., 1993), practice (Cordier et al., 1993), route (Seifert et al., 2017) and technique effects (Boschker and Bakker, 2002; Sibella et al., 2007). Furthermore, data collection to perform an entropy calculation is highly feasible involving use of a single camera (Sanchez et al., 2010). Figure 1 shows how entropy is calculated (Figure 1A) and with respect to how changing the length of an analyzed trajectory with the convex hull affects outcomes.

In climbing tasks, entropy outcomes are particularly increased when route difficulty is hard relative to the ability level of the climber, sometimes referred to as functional task difficulty (Guadagnoli and Lee, 2004), and, when the route has not yet been practiced (Cordier et al., 1994b). For example, when the functional difficulty of a route is increased, by modifying the number of choices embedded into it, entropy increases even in experienced climbers (Seifert et al., 2015). Practice effects have also been reported, with performance after repeated practice generally converging to an asymptote level at a rate dependent on the initial skill level of the climbers. Typically, the higher the initial skill level, the more rapid an asymptote is reached (Cordier et al., 1993, 1994a,b). Intriguingly, Boschker and Bakker (2002) found that prior knowledge about advanced inter-limb coordination patterns can improve entropy in beginners, allowing them to improve performance faster through practice (Seifert et al., 2013b).

Notably in Boschker and Bakker (2002), practice of less advanced techniques also resulted in entropy values similar to those observed when advanced actions were used, suggesting that the route designs may not have required more advanced technique for improved performance. Sanchez et al. (2010) also raised the concern, that, when elite climbers were compared on routes close to the limits of their ability level, no relationship between the climbers' performance GIE was shown. This may have been because the climbers had not practiced physically on the wall before testing began, and, the difficulty was close to the climbers' ability limits (Sanchez et al., 2010, p. 360). Implications of these studies suggest that, through observing repeated practice, larger learning effects can be expected when route difficulty is closer to a climber's ability level (Cordier et al., 1993). It is worth emphasizing, however, that these findings also indicate that in some cases a higher entropy may not necessarily indicate poor performance (Davids et al., 2014).

Aside from cases where task and skill interaction effects make entropy difficult to interpret, the variable is limited in other ways. Currently the application of GIE is limited to a single plane of analysis and important anterior-posterior plane translations (Sibella et al., 2007; Zampagni et al., 2011; Russell et al., 2012; Robert et al., 2013) or rotations around any given axis are missed (Seifert et al., 2014b, 2015). Of additional concern, is that if a climber is “blocked” at certain points in the climb, the GIE magnitude will only be influenced if there is an increase in the length of the trajectory during this time. For example. this can occur when postural readjustments are made. If no movement at the hip occurs during a stoppage, however, GIE magnitude will not be affected (Watts et al., 2016). Thus, changes in constraints, such as the use of a top-rope (a rope is secured to the top of the route prior to performance) vs. lead roping (where the climber needs to secure the rope to multiple fixed points during climbing for safety), may not lead to significant differences in entropy because they do not require a significant reorganization of the pathway taken through the route (cf. Hardy and Hutchinson, 2007). Nonetheless, GIE is a highly usable method, where limitations are made up for in ease of data acquisition and interpretation.

### Temporal indicators of fluency

Temporal measures interpreted with respect to continuity of climbing performance include the: (i) relationship between static and dynamic movements at the hips (Cordier et al., 1994a; Billat et al., 1995; Nieuwenhuys et al., 2008; White and Olsen, 2010; Seifert et al., 2013b, 2014c); (ii) relationship between hold grasping and moving between holds (Pijpers et al., 2005, 2006; Nieuwenhuys et al., 2008; White and Olsen, 2010); (iii) plateau durations at the hips (Seifert et al., 2013b, 2014c); (iv) within-route climb time (Sanchez et al., 2010; Draper et al., 2011b; Seifert et al., 2013a); (v) time spent in three-hold support (Sibella et al., 2007); and (vi), movement frequency (Cordier et al., 1996).

Quantifying the amount of time spent in different climbing-specific activity states provides one of the better temporal indications of the climbers' adaptations to route properties. For example, the degree of mobility is sensitive to local changes in the route's difficulty level, including crux and rest points (Sanchez et al., 2010), and can detect differences between individuals who fall or complete the route (Draper et al., 2011b). The most predominant approach to estimate performance in the temporal dimension is the computation immobility to mobility ratio, calculated by determining how long, with respect to the total climb time, an individual's COM or limbs remain in a stationary state relative to its moving state.

According to Billat et al. (1995) time spent immobile reflects time under isometric contraction, subsequently incurring an energy cost. However, since depending on the nature of the hand holds, this time can either increase fatigue in the finger muscles (Vigouroux and Quaine, 2006) or provide an opportunity to allow these muscles to recover (Sanchez et al., 2012), the characteristics of the route design needs to be addressed (for an innovative modeling approach see, Tosi et al., 2011). Indeed, it has been shown that periods of immobility can reflect strategic actions with respect to demands on the physiological system imposed by route design (Billat et al., 1995; White and Olsen, 2010). For example, different gripping techniques provide the possibility to vary the arm angle, which might afford more or less rest while grasping a hold and remaining immobile (Amca et al., 2012). This is also true in terms of the overall posture that climbers can adopt. For example, when sitting away from the wall with arms extended, passive forces can be exploited for remaining on the wall at a reduced energy cost (Zampagni et al., 2011; Russell et al., 2012).

Alternatively, White and Olsen (2010) also speculated that high immobility at the hip, in the case of bouldering, reflects an inability to perceive how to move through a route continuously, reducing performance in the activity. Sanchez et al. (2012) provided some evidence for this argument, showing that more experienced climbers spent longer periods at rest locations within routes when not given an opportunity to view the route from the ground. This finding suggests that immobility can indicate visual exploration of upcoming holds. Thus, individuals might benefit from periods of immobility at the hips and longer periods of reaching because exploratory actions might help to determine more effective pathways through the route (Nieuwenhuys et al., 2008; Sanchez et al., 2010; Seifert et al., 2015). Indeed, typically beginners show high levels of immobility, suggesting a lack of effective pick-up of information for perceiving climbing opportunities for route progression (Pijpers et al., 2005, 2006).

A key disadvantage of immobility is that classifying an individual as immobile is commonly undertaken by frame-by-frame analysis of an operator. For example, criteria for mobility have included statements like: “progress of the hips was observed” (Billat et al., 1995) whereas, criteria for static climbing have included: “no discernible movement in pelvic girdle” (White and Olsen, 2010). In an ice-climbing study, an automatic approach was taken by Seifert et al. (2014c) using a definition based on a movement threshold. In this case, immobility was considered when, along the vertical axis, pelvis displacement was less than 0.15 m for durations longer than 30 s. This approach, however, required manual digitisation of the hips and was limited to analysis of vertical displacement actions of ice-climbers. Similar problems arise when manually coding limb states, where a limb is determined as moving between holds (mobile) or is in contact with a support surface (immobile) (Pijpers et al., 2006; White and Olsen, 2010). Thus, since immobility is generally determined as the lack of displacement over time, directly using velocity is a possible solution suggested here. Specifically, for a trajectory *x* : [*O, T*] → *R*^3^, we find the threshold based immobility to mobility ratio as:

(3) $$\begin{array}{l} IMR_{x}=\frac{\sum_{i=1}^{N}P_{i}}{N} \end{array}$$

(4) $$\begin{array}{l} P_{i}=\{\begin{array}{cc} 1, & if\;v_{i}<thresh\\ 0, & if\;v_{i}\geq thresh \end{array} \end{array}$$

(5) $$\begin{array}{l} v_{i}=f\sqrt{x_{i}^{2}+y_{i}^{2}} \end{array}$$

Of additional concern when using immobility is that the (ir)regularity in the temporal dynamics of movements are not considered (Seifert et al., 2013b). For example, a climber could remain immobile at a single location on the wall, with the remaining climb time measured as mobile. Cordier et al. (1996), addressed this concern using a spectral dimension analysis of the last five practice trials (of 10) and showed that temporal movement dynamics of experts were periodic, since they displayed vertical displacement of the hips at regular intervals of 3 s. Furthermore, phase portrait analyses of each group revealed that skilled individuals displayed more regular movement characteristics (stable dynamics), whereas, intermediate climbers exhibited less predictable dynamics. These findings suggested that advanced climbers achieved a stable “coupling” between their coordination repertoire and the environmental features. The temporal analyses used with reference to their GIE analysis (Cordier et al., 1993, 1996), showed that, whilst the intermediate climbers achieved similar levels of GIE efficiency relative to the advanced group, they still required more training to improve efficient temporal dynamics. Indeed, the major limitation of spatial and temporal measures is that, although they provide important information in isolation, interpreting the nature of movement adaptions during climbing can be enhanced by considering these outcomes in combination (Draper et al., 2011a; Magiera et al., 2013; Seifert et al., 2013a; Laffaye et al., 2014).

### Multi-variate approaches to understanding climbing fluency

**Figure 2:**
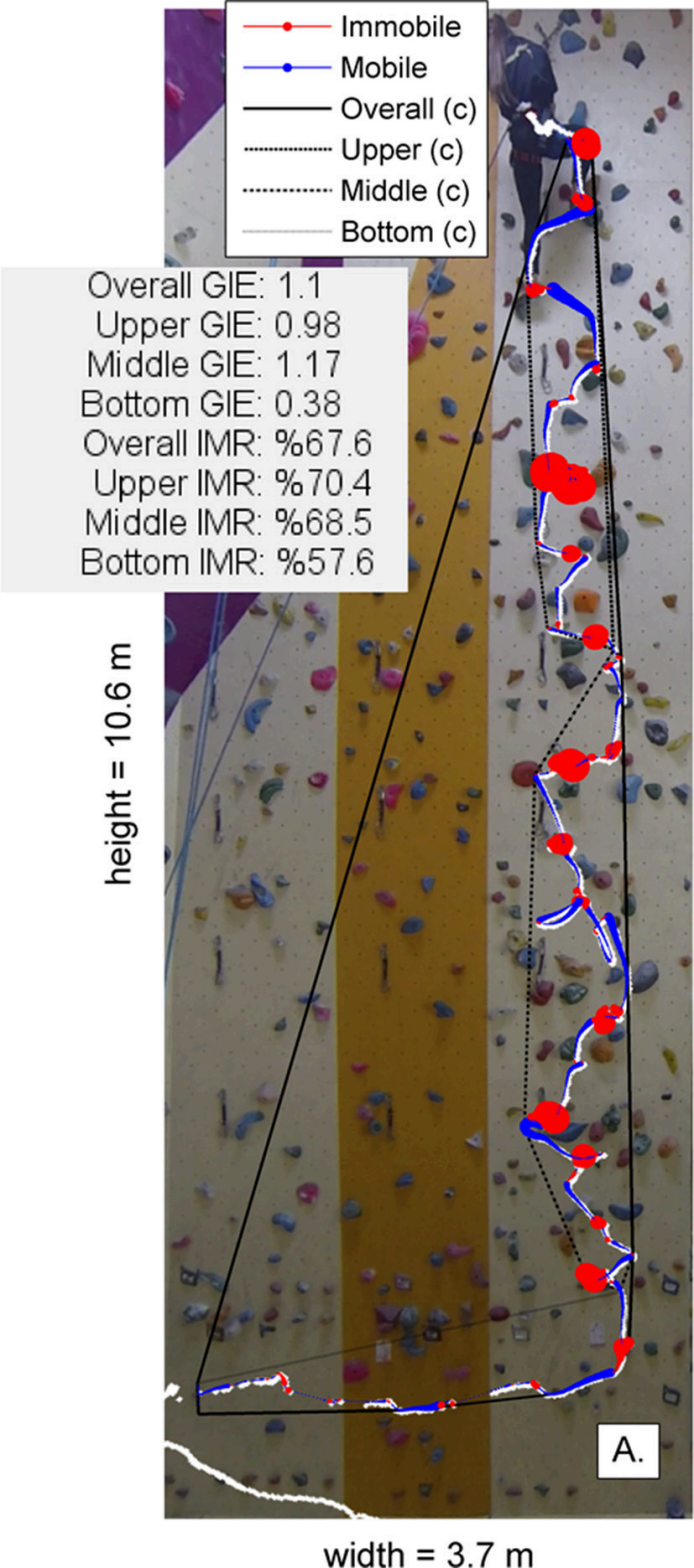
The relationship between entropy and immobility as a function of wall position. Radius of each point was scaled to increase in proportion to the duration spent in a given state (thus the larger the dot, the longer the individual was in the given state of mobility (i.e., blue line) or immobility (redline). c, convex hull. Also note that upper means the top convex hull. Middle means the middle convex hull, bottom means bottom convex hull, and overall means the total convex hull. GIE, geometric index of entropy; IMR, immobility to mobility; m, meters.

Thus, we now consider in more detail how combined measures of spatial-temporal indicators of performance can improve interpretation of climbing performance behaviors using exemplary data (Orth et al., 2014). In Figure 2, both immobility (using Equations 3–5) and GIE (Equations 1, 2) are calculated on a climbed trajectory at three sections of a beginner level route (French rating scale of difficulty = 5a). It is shown that, depending on which section of the route the climber is in, the relationship between GIE and immobility can be inversed. Indeed, spatial and temporal properties of behavior are probably co-adapted depending on the constraints on performance (Billat et al., 1995). For example, when required to use complex movements, such as when using dynamic moves, a high degree of mobility is probably also important. Conversely, when using less dynamic movements, a low level of mobility may help maintain a degree of stability, particularly when needing to keep the COM close to the wall (Fuss et al., 2013). If co-adaptation between GIE and IMR do support efficient climbing, a clear hypothesis is that immobility and movement complexity are co-adapted to maintain performance in terms of smoothness or jerk (Seifert et al., 2014b, 2015).

An important limitation in understanding the results related to performance fluency such as, Jerk, GIE, and IMR, is that, without a consideration of the climbers intentions during periods of immobility or increased entropy, these data may be mistakenly concluded as dysfunctional (Seifert et al., 2014b). The study by Fryer et al. (2012) illustrates this point nicely. In this study more experienced climbers exhibited a *greater* percentage of time spent immobile, compared to less experienced individuals. After carrying out an activity analysis into the types of actions undertaken during rest, it was found that the more experienced climbers were actively resting during immobility, either applying chalk to their hands or shaking their hands. In this example, without additional data from the activity analysis, it may have been erroneously concluded that the climbers were stopping more due to the greater physiological demand imposed by the route. In actual fact, the data highlighted the climbers' self-management of their internal states, relative to their exploitation of opportunities for rest in the climbing route, an important skill-dependent performance behavior (Fryer et al., 2012). This case exemplifies how interpreting activity states of climbers can provide mechanistic insights on fluency measures (Seifert et al., 2013b, 2015).

## The role of activity states in climbing for understanding performance

It is generally assumed that the task goal corresponds to the intentions of the individual where in climbing, the goals of the task are to: (a) not fall; (b) get to the end of a route, and (c), use an efficient pathway and movement patterning that reduces prolonged pauses (Orth et al., 2016). However, importantly, intentions can be influenced by skill (Rietveld and Kiverstein, 2014), which are reflected in adaptations that emerge with respect to dynamic constraints (Balagué et al., 2012; Davids et al., 2015). Thus, estimates of the intentions of individuals during performance can help place performance outcomes more accurately in line with what an individual was trying to achieve.

Seifert et al. (2014c), for example, showed that expert ice-climbers went about achieving their intentions to maintain energy and economy by focusing perception and action toward specific intentions. Actions were related to the perception of information for the usability of existing holes in the ice fall in so far that they tended to seek holds that did not require them to swing their ice tool. In contrast, the intentions of inexperienced climbers pertained to stability, where perceptions were focused on information related to the size of holes in the ice surface. In this case actions were motivated for achieving deep, secure, anchorages during ascent. Indeed, inexperienced climbers displayed significantly longer periods of immobility at the hips, higher amounts of swinging actions prior to making a definitive anchorage with their ice-tools, and tended to adopt a “X-like” body position with the arms and legs spread out for stability. Whilst the inexperienced climbers showed poor performance in terms of temporal fluency, their exploratory actions were in correspondence to the key intention to avoid falling. Thus, the distinction between exploratory and performatory actions is fundamental to understanding climber intentions and the functionality of their actions during performance and learning.

### Performatory actions

According to Pijpers et al. (2006), performatory actions are meant to reach a specific goal and include: moving a hand or foot from one hold to the next to use it as support for further climbing actions (Pijpers et al., 2006; Nieuwenhuys et al., 2008; White and Olsen, 2010); using a hold to move the entire body vertically or ascend the route (Sanchez et al., 2012; Seifert et al., 2013a, 2014c); using a hold to support recovery actions (Fryer et al., 2012; Sanchez et al., 2012); and, making visual fixations during movement at the hips (Nieuwenhuys et al., 2008). Theoretically, performatory actions correspond to actions that are intended for progression. If performatory actions are effective they should improve fluency, by reducing the amount of time spent immobile and contributing to ongoing progression through the route. For example, a climber might skip holds, use a more difficult movement (Sibella et al., 2007) or use less advanced actions (Boschker and Bakker, 2002) which might result in more or less fluid climbing performance.

### Exploratory actions

Exploratory actions, on the other hand, are primarily information gathering movements (Pijpers et al., 2006) where the type of information important to support perception of movement opportunities on a surface (i.e., affordances) can pertain to modalities such as haptic, auditory, visual and kinesthetic (Smyth and Waller, 1998; Seifert et al., 2014c). Exploratory actions have included: when climbers explore whether a hold is within reach (Pijpers et al., 2006); when a hold is touched without being used as a support (Pijpers et al., 2006; Nieuwenhuys et al., 2008; Sanchez et al., 2012; Seifert et al., 2013b, 2014b,c); when an anchorage is weighted to test its fallibility (Seifert et al., 2014c); when tools are used to swing without a definite anchorage (Seifert et al., 2011, 2013a, 2014c); and when a visual fixation occurs whilst an individual is immobile (Nieuwenhuys et al., 2008). An increase in exploratory indices is generally associated with poorer performance on measures of fluency (Orth et al., 2016). For example, if a climber stops because they cannot perceive an effective path through the route (Cordier et al., 1993; Sanchez et al., 2012), this would be associated with a higher frequency of hold exploration (Pijpers et al., 2006) and possibly an increased GIE (Cordier et al., 1994b).

However it is important the functionality of exploration, since as exploration reduces, fluency can improve (Seifert et al., 2013c, 2014b), suggesting an important relationship between exploration and performance improvement through practice. For example, Seifert et al. (2015) recently showed how exploration remained elevated under transfer conditions after a period of variable practice (i.e., where each training session involved practice on one of three different routes). In this study, implications were that potential mechanisms underpinning the positive transfer in climbing were related to the efficient use of exploration.

## Variability in activity states and their functionality

In this final section, we explore some of the implications of linking different activity states with performance outcomes (summarized in Table 2) with predictions for future work. Specifically, we attempt to explain the goals or intentions underpinning behavioral variability related to both activity state and spatial-temporal measures. Indeed, a key outcome of this review has been the identification of a broad range of activity states that have been reported in the literature as potentially important for performance during climbing. As clarified in Table 2, key activity states include: immobility; postural regulation; grasping; grip change; active recovery; reaching; reaching and withdrawing; traction; and, chaining movements in succession.

**Table 2:** Relationships between spatiotemporal outcomes, discrete actions and climbers intentions.

**Activity state**	**Limb activity (A) combined with spatial (GIE) and temporal (IMR) outcomes**	**Function (individual intentions)**
Immobility	A: All limbs stationary and: 1. IMR ↑ and GIE ↓	1. Passive recovery (Seifert et al., 2013a); Visually explore (Nieuwenhuys et al., 2008; Sanchez et al., 2012); establish base of support.
Active recovery	A: 1 limb moving and behind the body: 1. IMR ↑ and GIE ↓	1. Relieve the forearms, apply chalk (Fryer et al., 2012); Visually explore (Sanchez et al., 2012).
Postural regulation	A: All limbs stationary and: 1. IMR ↓ and GIE ↑ 2. IMR ↓ and GIE ↓	1. Exploration of different body orientation(s) (Cordier et al., 1993, 1996; Seifert et al., 2015). 2. Use of different body orientation(s).
Grasping	A: 1 limb moving and: 1. IMR ↑ and GIE ↓	1. Preparation for hold use (Fuss and Niegl, 2008; Boulanger et al., 2016).
Grip change	A: 1 limb moving and: 1. IMR ↑ and GIE ↑	1. Explore hold grasp technique (Boulanger et al., 2016).
Reaching	A: 1 limb moving and: 1. IMR ↑ and GIE ↓	1. Change holds.
Reach and withdraw^**^	A: 1 limb moving and: 1. IMR ↑ and GIE ↓ 2. IMR ↑ and GIE ↑	1. Efficient exploratory reach (Seifert et al., 2015). 2. Inefficient exploratory reach (Seifert et al., 2015).
Traction	A: ≥1 limb moving and: 1. IMR ↑ and GIE ↓ 2. IMR ↓ and GIE ↑	1. Movement using face-on body position (Fuss et al., 2013). 2. Movement with body roll (Fuss et al., 2013).
Chaining movements in succession	A: ≥1 limb moving and: 1. IMR ↓ and GIE ↓	1. Fluent performance (Cordier et al., 1996).

*IMR ↑, means the individuals is more immobile; IMR, ↓ means the individual is more mobile; GIE ↓, means the movement is less complex; GIE ↑, means the movement is more complex*.

** *Requires that the next state is not a lifting state*.

Typically, total immobility is a sign of poor performance (e.g., being “blocked”). However, functional movement variability can be identified. Postural exploration is probably particularly relevant for beginners, as this may allow an individual to determine more efficient positions and new body-wall orientations that may be important for more advanced movements (Seifert et al., 2015). Another possibility discussed has been that the individual may benefit from immobility by visually exploring upcoming holds, perhaps indicated by the amount of fixations made and their relative distance to the individual during immobility (Sanchez et al., 2012).

Exploration can also include reaching to touch a hold but not grasping it or using it to support the body weight (Seifert et al., 2015). This is probably important for perceiving accurate body-scaled actions (Pijpers et al., 2007). Perhaps, as different techniques, such as dynamic moves (Fuss et al., 2013), become part of an individual's action capabilities, this boundary of reachability may distinguish individuals of different skill levels. Making adjustments in how a hold is grasped prior to using it is also a form of exploration in terms of its “grasp-ability” affordance. Prior to applying force to a hold climbers can be seen, in some cases, to make adjustments to how they position their hand on a hold. Such exploratory actions may be important to improve the amount of friction that can be applied to the hold (Fuss et al., 2013), or, to enable a qualitatively different way of using the hold such as in cases where multiple edge orientations are available (Seifert et al., 2014b).

Finally, It has been argued that exploration can support perception of affordances or opportunities for new climbing moves (Seifert et al., 2013c). This may be observed by examining how climbing actions differ through practice. For example, over repeated attempts, different route pathways, body orientations or grasping patterns might be used, reflecting exploration emerging during learning. Thus, during interventions, the nature of learning behavior, in so far that it can be related to the progression toward higher levels of performance (or fluency), may be better understood by evaluating the level at which exploration emerges.

A substantial challenge, in future research is in measuring exploration at different levels of analysis with respect to performance, both, in technically manageable and theoretically consistent ways (Seifert et al., 2014d; Orth et al., 2016; Schmidt et al., 2016). For instance, whilst, performatory and exploratory actions are predominantly assessed by considering overt action at the limbs, such characteristics are distinguishable across other levels, such as overall organization of the body (Russell et al., 2012; Seifert et al., 2014a), postural regulation (Boulanger et al., 2016), visual search (Nieuwenhuys et al., 2008) and at more refined levels of control at hand-hold interaction (Fuss and Niegl, 2008). Identification of these movement features is a clear research challenge for future work.

In particular the role of exploration for improving transfer is worth more attention. Indeed, any on-sight climb (where a climber attempts to climb a route they have never physically practiced) might be conceptualized as a skill transfer problem, requiring adaptations during performance to unfamiliar surface properties and in dynamic environments (such as outdoors). Assuming positive transfer (Carroll et al., 2001; Issurin, 2013) is supported by the ability to skillfully seek efficient route pathways and climbing opportunities, interventions aiming to improve performance on new routes should consider the functional role of exploration during practice.

## Conclusion

This review has demonstrated the importance of relating fluency and activity measures for understanding climbing actions and performance outcomes. Whilst numerous variables have been reported across the extant literature, many of these fail to capture how climbers adapt to a route whilst climbing. We have argued that there should be an emphasis on considering spatiotemporal measures concurrent with the evaluation of climbing specific activity states. Depending on the level of detail, such states can include: immobility; postural regulation; grasping; grip change; active recovery; reaching; reaching and withdrawing; traction; and, chaining movements in succession. In doing so, a more comprehensive picture of how climbers successfully adapt to a given route can be taken. In particular, the climber's intentions should be easier to estimate. For example, by combining these data, it is possible to more accurately determine whether an individual is stopping in order to recover or because he/she cannot perceive opportunities for progressing. We have also highlighted limitations in traditional performance measures (i.e., entropy and immobility). If activity analysis is not feasible, the main recommendation is that entropy and immobility should be concurrently assessed with respect to jerk. In doing so, the efficiency with which a climber is able to co-adapt movement complexity with required mobility can be addressed.

For future research, there is a major lack of understanding for how climbers transfer their skills to new routes and warrants more innovative approaches. Skill transfer is an essential part of climbing and indeed physical activity and sports in general. We anticipate that more successful climbers are more effective in how they explore new routes. Thus, characterizing how exploration is functional to climbers, and how they learn to explore effectively, such as based on practice constraints that require exploration, is a key problematic for future work.

## Practical summary

Findings in this review have a number of practical implications.

- Skilled performance in climbing can be broadly characterized in terms of how fluently an individual reaches the end of the route. Measures of jerk (at the body's center of mass) currently provide the best global indicators of fluency. Skilled climbers maintain fluency under changing constraints by adapting both how fast they climb (their level of mobility) and the complexity of their movements. The clearest indication of these adaptive behaviors is in experiments involving modification of hold size, the orientation of holds and the number of ways a hold can be used. In cases where holds require more complex body positions, experienced climbers will increase both how fast they climb and how complex their movements are.
- In this review we stressed that skilled climbers explore efficiently—this allows them to adapt strategies, while climbing, to help maintain performance. Indeed, efficient exploration would seem essential for effective performance under on-sight conditions. Constraints that support learning that improves on-sight performance, may be achieved by promoting exploration during practice. Recommendations are that the transition toward skilled behavior involves learning efficient exploration across different levels (i.e., using hands/feet, limb, varied hip orientations and visual inspection). Learning design should support each learner's current needs (such as maintaining stability or improved performance). Practitioners have an abundance of strategies at their disposal—what is essential is that movement variability, such as exploration, can be viewed as functional to the individual.
- These findings also provide practitioners with a way of assessing learning. Currently, automatic tracking procedures of the climber's hips and limbs *en route* presents an opportunity to assess learning. For instance, the rate of learning on new routes may be indexed as the rate at which jerk stabilizes or the rate that exploration subsides. In facilitating learning, the practitioner should constrain information during practice that can be meaningful to the climber for future performance and practice contexts. We suggest designing practice tasks to influence affordance perception. That is, to manipulate constraints so that the individual is challenged during practice to seek out movement opportunities relevant to completing a route fluently. Consistent with the findings in this review, early in learning or under challenging circumstances, affordance perception provides a means of remaining fixed to the surface. Later in learning, a climber perceives affordances for linking movements in a more periodic and fluent manner, supporting efficient and effective performance.

## Author contributions

DO, GK, KD, and LS conceived, performed and wrote the review in collaboration.

### Conflict of interest statement

The authors declare that the research was conducted in the absence of any commercial or financial relationships that could be construed as a potential conflict of interest.
